# A test of construct isomorphism of the Belief in a Zero-Sum Game scale: A multilevel 43-nation study

**DOI:** 10.1371/journal.pone.0203196

**Published:** 2018-09-28

**Authors:** Joanna Różycka-Tran, Guido Alessandri, Paweł Jurek, Michał Olech

**Affiliations:** 1 Institute of Psychology, University of Gdansk, Gdansk, Poland; 2 Department of Psychology, Sapienza University of Rome, Rome, Italy; Coventry University, UNITED KINGDOM

## Abstract

**Background:**

We examined the equivalence of the individual and the country-level factor structure of the Belief in a Zero-Sum Game (BZSG) scale, a tool designed to measure antagonistic beliefs about social relations (i.e., perceived social antagonism) in the struggle for limited resources.

**Aims:**

In this article we focused on a test of construct isomorphism in a multilevel modeling approach. It was hypothesized that the BZSG measure satisfies all requirements for a strong level of configural isomorphism, and thus that it is useful to investigate BZSG at both the individual and the country levels. The relationships between the BZSG at a country level with other macro-socio-economic indicators were also investigated.

**Method:**

Multilevel confirmatory factor analysis (MCFA) was carried out on a cross-country sample composed of 11,368 participants from 43 different countries. We also used the country-level latent BZSG factor in each country as an indicator of a property that we attributed to a particular culture: cultural dimension (collectivism-individualism), macroeconomic indicators (GDP per capita and GNI per capita) and macrosocial indicators (Human Development Index and Democracy Index) describing societies.

**Results:**

The results revealed an isomorphic factor structure of perceived social antagonism (measured by BZSG scale), defined in terms of the equivalence factor structure at the both individual and country levels. Furthermore, the relationship between the perceived social antagonism, gross national income per capita, and collectivism were confirmed.

**Conclusions:**

Our study supports the usefulness of the BZSG scale for cross-cultural comparison, in the case of its isomorphic structure. At the country level, antagonistic beliefs emerge in hierarchical collectivist societies with lower income. The main contribution of this article is the presentation of the test of construct isomorphism. We made an effort to present a full perspective on construct isomorphism putting together two different but very recent approaches.

## Introduction

### Multilevel cross-cultural approach

Hofstede [1] claims that culture is shared among individuals, which statistically means that people are nested within culture. However, in previous large cross-cultural studies, Hofstede’s approach was based only on the country-level analysis, because Hofstede identified four value dimensions at the country level but did not find matching dimensions at the individual level. From the other side, Schwartz and Bilsky [2] proposed a value-based model presented at the individual level. Even Schwartz [3] discriminated different sets of value constructs at individual and country levels (ten individual value types versus seven cultural values); this was based on separate analyses per level. Regardless of these single-level approaches, individual- and country-level data should be analyzed simultaneously via multilevel models [4,5].

In addition to axiological values [3], some epistemological belief constructs seem to exist at both individual and country levels; these are called social axioms, defined as “generalized beliefs about oneself, the social and physical environment, or the spiritual world, which form an assertion about the relationship between two entities or concepts” ([6], p. 289). Social axioms are proposed as fundamental psychological constructs tapping a person’s beliefs about the social world [7]; they help their holders to navigate social situations [8]. Bond et al. [9], analyzed the factor structure of social axioms at both the individual and country level, using data collected from 41 cultures; where *soci(et)al cynicism* appeared as an *isomorphic* axiom, i.e., was equivalent across both levels [10–12].

Another recently identified social axiom is a “general belief about the antagonistic nature of social relations, shared by people in a society or culture and based on the implicit assumption that a finite amount of goods exists in the world, in which one person’s winning makes others the losers” ([13], p. 526). Stemming from game theory [14], it appears that there are relatively permanent convictions that social relations are like a zero-sum game (i.e., antagonistic), which can be measured using the Belief in a Zero-Sum Game (BZSG) scale [13].

### Belief in a zero-sum game (BZSG) scale

To measure the belief that life is conceived as a zero-sum game (i.e., perceived social antagonism), the BZSG scale was developed [13]. The tool consists of eight items reflecting beliefs about antagonistic competition over scarce resources. This scale was first used in a study on a Polish national sample (*N* = 1,133), and psychometric properties of the BZSG scale have already been described [15]. Moreover, several experimental and correlational studies have been performed with different samples, which found that the BZSG correlates with a host of judgmental and behavioral variables [13,16].

Next, the reliability and validity of the scale was investigated across 37 countries (*N* = 6,138) [13], and finally the measurement invariance of the one-factor 8-item BZSG scale was supported at the individual level (*N* = 9,907) across 36 countries in multigroup confirmatory factor analysis (MGCFA) [16]. Although previous results have confirmed the measurement invariance of the BZSG scale, the cross-level equivalence (i.e., a test of construct isomorphism) has not been investigated.

Based on Cheung et al. [4], who evaluated the individual- and country-level structures of social beliefs, we were interested in the cross-level equivalence of the BZSG scale measuring perceived social antagonism in relations.

### Individual- and country-level of the BZSG scale

Based on previous findings, we suggest that BZSG appears as a personal worldview and as a cultural ideology in a given country. Whereas at the individual level, people fight over limited resources, similar phenomena are encountered at the country level between groups and nations. At the individual level, the 37-nation study (with a different sample) found that people who believed that social relations are like a zero-sum game tended to trust others less, have lower self-esteem, and perceive their economic status as lower than others [13]. At the country level, it was found that BZSG was more prevalent in countries with lower income. Although other correlates of BZSG were found, such as collectivism, social development, or democracy, only collectivism (positive relationship) and income (negative relationship) proved to be significant predictors within multilevel modelling [13].

Taking these results into account, it seems that BZSG emerges in hierarchical collectivist societies with an economic disparity of scarce resources. Economically deprived countries are more likely to view the social world as an arena for a fierce fight over the limited amount of wealth and are also more susceptible to conflicts [17]. However, neither collectivism nor national income were investigated in the multilevel factor model BZSG that included country-level variables. Such an investigation would be useful for describing the vulnerable conditions for shaping antagonistic beliefs and behavior within individuals.

We postulate that antagonistic beliefs about social relations (i.e., perceived social antagonism measured by the BZSG scale) is an isomorphic axiom, which influences how individuals or nations perceive their relationship in situations where their interests are interdependent in the context of limited resources. In other words, we assume that the BZSG scale operates isomorphically at the individual and country level of analysis, namely that it manifests as the same latent construct (is measured in the same way) at both levels (hypothesis 1).

The confirmation that the BZSG scale measures the same construct at both the individual and the country level, allows for using the country-level latent BZSG factor scores in each country as an indicator of a property that we can attribute to a particular culture. In other words, we expect to confirm that the BZSG measure satisfies all requirements for a strong level of configural isomorphism, and thus that it is useful to investigate BZSG at both the individual and the country levels. We start by testing configural isomorphism, computing the fit of a unidimensional model for the BZSG scale, where the hypothesis of BZSG as an isomorphic construct was formally tested by performing specific tests of construct isomorphism [18].

Based on previous findings, we also assumed the negative relationships between the BZSG scale at a country level with objective macro-socio-economic variables such as: individualism, a country’s standard of living (measured by GDP per capita, GNI per capita, and Human Development Index), and a country’s state of democracy (measured by the Democracy Index; hypothesis 2). In other words, we expect that citizens share antagonistic beliefs about social relations to a lesser extent in more individualistic countries with a higher standard of living and a higher level of democracy compared to more collectivistic countries with less wealth and a lower level of democracy. In individualistic countries, people are expected to be independent in acquiring resources, while in collectivist countries the distribution of goods is associated with belonging to the group. We can assume that in highly developed countries, in which citizens are more salaried, their needs are more satisfied. So, antagonistic beliefs about social relations shared in such a society are weaker.

### Testing construct isomorphism

Several methodologies have been proposed for investigating different aspects of the broad issue of measurement invariance [19] or measurement equivalence [20]. One set of methodologies addresses a specific aspect of measurement equivalence, namely construct isomorphism (see [21,22]), and points to structural identity at the different levels. Therefore, differences between individuals and differences between countries on psychological values can be explained in terms of the same concepts or dimensions. In other words, the psychological nature of human individuals determines the structure of beliefs or values, where aspects of societies (country-level factors) can have an indirect impact on these individual-level factors, but do not influence the belief or value structure [18]. While individuals have their own beliefs about social relations, the beliefs that are shared are identifiable at the country level of analysis. This is an example of ideal form of bottom-up processes, which is rooted in a theoretical model of isomorphism, where lower-level data are assumed to be similar to the higher-level construct [23,24].

Psychometrically, measurement invariance across different levels of analysis is called isomorphism [22,25,26]. According to Tay et al. [22], when the same number of factors and the same pattern of free and fixed loadings exists at the individual and at the country level, strong configural isomorphism is said to occur. When the same number of factors is ascertained at the individual and country level, but the pattern of fixed and freed loadings is different, there is a condition of weak configural isomorphism. When none of the above conditions occur (different number of factors, different pattern of fixed and free loadings), there is no evidence of isomorphism. According to Jak [21], strong construct isomorphism can occur in two forms. In the first stronger type, construct isomorphism entails both: (1) equality of factor loadings across levels of analysis, and (2) zero residuals at the higher level of analyses (in our case, the country level). As shown by Jak, Oort, and Dolan [27], this model translates to a multiple group model assuming strong factorial invariance across countries holds, and attests that the items composing the BZSG scale have the same interpretation across levels. Loosely speaking, no cause other than the common factor is exerting an influence on the observed indicators. Another, less restrictive form of construct isomorphism, instead requires an equality constraint on loadings across levels but lets the residual at the higher level be freely estimated. Interestingly, when using this model the causes of these residuals can be investigated by adding external variables to the model.

Because one of the challenges for cross-cultural psychology is not only to ascertain the equivalence of the construct meaning and measurement, but also to identify structures of psychological constructs both at the individual and country levels [18], we conduct a test of isomorphism of the BZSG scale across levels.

## Materials and methods

### Participants and procedure

Data were collected from 11,368 participants in 43 countries at two time points. The first wave (*N* = 5,158, 37.1% men) [13] consisted of student samples from 30 countries. The mean age of students was 21.25 years (*SD* = 4.71) and they were studying social sciences or business. The second wave (*N* = 6,210, 39.5% men) ([16] five countries were added as a new data) consisted of student samples from 30 countries with a mean age of 21.54 years (*SD* = 4.80), and the distribution of academic affiliations was similar to that of the first wave. Seventeen countries contributed samples in both waves (Belgium, Brazil, Bulgaria, China, the Czech Republic, the United Kingdom, Hungary, India, Japan, Poland, Portugal, Russia, Serbia, Slovakia, South Africa, Spain, and Vietnam), so that these two phases allowed us to put together 60 samples from 43 countries.

All samples were composed of college students, recruited as volunteers, some of whom received course credit for participation. The protocol of this study was approved by the Ethics Board for Research Projects at the Institute of Psychology, University of Gdansk. According to the local law of different universities, no written permission from participants was required, as data were collected and analyzed anonymously. Participants were assured that their data would remain anonymous and confidential. However, we followed APA standards and the Declaration of Helsinki during data collection. Information on sample composition is provided in Table 1. Participants completed a paper and pencil version of the BZSG scale while also reporting age and sex.

**Table 1:** Samples composition, Cronbach’s alphas and descriptive statistics of BZSG in 43 countries.

			Age		BZSG		
Country	N	% of men	M	SD	Cronbach’s alpha	M	SD
Armenia	220	52.3	19.00	1.16	.83	3.61	1.31
Azerbaijan	117	37.6	20.84	1.88	.89	3.66	1.17
Belgium	478	15.7	19.06	2.96	.83	3.29	1.03
Brazil	324	47.2	22.38	6.72	.77	3.22	1.03
Bulgaria	301	29.2	23.15	5.20	.86	3.48	1.18
Canada	83	72.3	22.84	2.16	.85	3.60	1.22
China	467	41.8	20.68	2.21	.82	4.07	1.03
Colombia	140	50.0	19.09	3.98	.89	3.32	1.27
Czech	345	28.4	24.06	7.68	.82	2.89	.87
Dominican	100	52.0	22.65	5.71	.69	4.13	1.02
Estonia	303	29.7	23.62	7.07	.90	3.42	1.12
Finland	102	14.7	23.45	5.35	.80	3.37	.83
Georgia	100	21.0	20.18	2.76	.70	3.99	.97
Germany	296	16.2	22.83	8.04	.80	3.31	.96
Honduras	108	14.8	22.61	4.47	.73	3.89	1.15
Hungary	311	30.5	21.23	1.99	.84	3.29	1.07
India	304	41.1	22.73	1.75	.82	4.14	1.22
Indonesia	200	50.0	21.38	1.65	.88	3.53	1.26
Iran	199	50.3	21.31	1.51	.78	4.25	1.49
Israel	125	24.8	24.03	2.24	.89	2.93	1.05
Japan	413	57.9	19.90	2.24	.84	3.78	1.11
Latvia	161	32.3	29.02	9.59	.95	4.24	1.26
Mexico	228	50.4	22.55	4.96	.87	4.08	1.46
Nepal	190	51.1	22.64	4.43	.74	4.26	1.17
Pakistan	200	51.0	21.49	1.60	.86	3.89	1.31
Panama	176	66.5	21.63	5.71	.85	3.51	1.25
Philippines	108	34.3	16.75	1.34	.80	3.88	.97
Poland	448	36.8	22.00	3.41	.90	3.29	1.15
Portugal	264	34.5	21.77	6.11	.87	3.42	1.15
Puerto Rico	299	57.2	20.25	2.23	.84	3.56	1.29
Romania	207	51.2	21.49	4.25	.90	3.83	1.32
Russia	397	15.1	20.27	2.72	.86	3.15	1.04
Serbia	400	44.3	22.39	5.74	.86	3.82	1.25
Singapore	108	42.6	20.97	1.93	.84	3.99	.91
Slovakia	386	27.7	21.26	1.65	.83	3.48	.94
South Africa	369	53.4	19.92	2.40	.85	3.72	1.18
South Korea	211	45.5	22.19	1.92	.89	3.93	1.03
Spain	330	35.5	20.40	4.88	.85	3.92	1.17
Taiwan	298	67.8	21.55	2.49	.80	4.34	.95
United Kingdom	466	18.9	19.73	2.60	.87	3.55	1.05
Ukraine	301	29.6	18.71	1.57	.76	4.16	1.02
USA	444	33.6	23.01	9.12	.87	3.30	1.06
Vietnam	341	46.0	20.44	2.34	.76	4.20	1.09
**Total**	**11,368**	**38.4**	**21.41**	**4.76**	**.85**	**3.66**	**1.17**

### Measures

#### Belief in a zero-sum game

The BZSG scale [13] consists of eight items (e.g., “Life is so devised that when somebody gains, others have to lose”, “The wealth of a few is acquired at the expense of many”). These items all reflect beliefs about antagonistic competition over scarce resources. The BZSG scale was translated into 20 languages (Armenian, Bulgarian, Chinese, Czech, Estonian, Flemish, French, Georgian, German, Hungarian, Japanese, Polish, Romanian, Serbian, Slovakian, Portuguese, Spanish, Russian, Ukrainian, and Vietnamese). National versions of the scale were created by bilingual individuals working in psychology or at the university using the back-translation procedure with the English version as the basis for all translations. In the first wave we used a seven-point scale (from 1 = *strongly disagree* to 7 = *strongly agree*), whereas in the second wave we used a six-point Likert scale (from 1 = *strongly disagree* to 6 = *strongly agree*). The second wave was a part of a larger project using various tools, so it was necessary to unify the scale of responses in that project. Linear equations were used to transform the six-point scale scores into seven-point scale scores. Before transforming and combining the data, we tested scalar (strong) invariance of both measurements (see [16]).

Participants completed a paper and pencil version of the BZSG scale as a subjective measure. To examine the relationship with macro indices, we used objective measures, such as:

#### Gross domestic product (GDP) per capita and gross national income (GNI) per capita

GDP per capita was calculated as the aggregate of all final goods and services produced within a nation in a given year divided by the population size; while GNI per capita was the value of final income earned by a country's residents (divided by country's population) including income earned abroad. Thus, GDP measures production, while GNI measures pure income. We used GDP per capita and GNI per capita in 2015, converted at market exchange rates to current U.S. dollars from statistical database compiled by the United Nations Statistics Division [28].

#### Human Development Index

The Human Development Index (HDI) is a summary measure of average achievement in key dimensions of human development: a long and healthy life, being knowledgeable, and having a decent standard of living. The health dimension is measured using life expectancy at birth. The education dimension is measured using mean years of schooling for adults aged 25 years or older and expected years of schooling for children of school-entering age. The standard of living dimension is measured using gross national income per capita. The HDI uses the logarithm of income to reflect the diminishing importance of income with increasing GNI. The scores for the three HDI dimensions are then aggregated into a composite index using the geometric mean [29]. We used data from 2015 available in Human Development Reports compiled by the United Nations Development Programme [29].

#### Democracy Index

The Economist Intelligence Unit’s Democracy Index is based on five categories: electoral process and pluralism, civil liberties, the functioning of government, political participation, and political culture. The five categories are interrelated and form a coherent conceptual whole. The condition of having free and fair competitive elections, and satisfying related aspects of political freedom, is clearly the basic requirement of democracy [30]. We used data from 2015 available in a report compiled by The Economist Intelligence Unit [30].

#### Collectivism-individualism

Collectivism, as a cultural dimension, represents a preference for a tightly knit framework in society in which individuals can expect their relatives or members of their in-group to look after them in exchange for unquestioning loyalty. Its opposite, individualism, can be defined as a preference for a loosely knit social framework in which individuals are expected to take care of only themselves and their immediate families [31]. We used scores on the collectivism-individualism dimension based on Hofstede et al.’s [31] studies. The data originates from different years, and was collected for 76 countries, partly based on replications and extensions of the IBM study on different international populations and by different scholars.

### Analytic strategy

To investigate the isomorphism of the BZSG scale across the individual and country levels, we conducted MCFAs in several successive steps following recommendations by Byrne and van de Vijver [32], who provided a procedure for the test of multilevel equivalence, analogous to the strategy used in multigroup modeling. In Step 1, a single-level CFA with robust maximum likelihood estimation and standard errors adjusted to account for cluster sampling (i.e., nested data) was used to establish the fit of the hypothesized one-factor model to the overall sample. This single-level CFA was performed on the variance/covariance matrix of the observed items that blurs the country and individual level of analysis. Then, we computed Muthén's [25] intraclass correlation coefficient (ICC) for each of the eight items of the BZSG scale. The ICC is calculated from a ratio of the maximum likelihood estimates of the latent within and between variance components, assuming random level effects. It ranges in value from 0 to 1, with higher values of the ICC indicating greater proportions of between-level variance and thus, greater bias if the multilevel nature of the data is not taken into account. In general, published studies used an ICC value of .10 as a cut-off criterion to conduct MCFA [26,33].

In detail, in Step 2 we investigated the fit of the one-factor model for the BZSG at the individual level, by letting the country level variance/covariance matrix be unstructured. In Step 3, we retested the fit of the one-factor model at the country level while letting the pooled individual-level variance/covariance matrix be unstructured. Then, in Step 4, we investigated the configural invariance of the BZSG scale across the individual and country level, by performing an MCFA based on the results of the previous steps, with: (1) loadings at the individual and at the country level, and (2) residuals at the between level freely estimated.

Finally, in Step 5, following guidelines from Jak [27], we investigated strong construct isomorphism by fitting a model with equal factor loadings across the within- and between-country level and zero residual variance at the country level to the data. Depending on the results offered by this model, we will estimate a further model similar to the one described above, but without the constraint of zero residual variances at the between-countries level (Step 6). Differently from the previous model, this latter model allows for country-level differences that the common factors do not account for (captured by significant item residuals), and thus allow us to model measurement bias across countries. Following Jak [34], we will use parameter estimates obtained under this model to estimate the degree of country bias affecting the individuals’ responses to the BZSG items.

In the last step (i.e., Step 7), we will use the best fitting model resulting from the above described analytical steps to investigate country-level differences in the common factor means, or BZSG, determined by between-country differences in GNI per capita, GDP per capita, HDI, the Democracy Index, and the collectivism-individualism dimension. We also used the abovementioned measures for exploring potential sources of measurement bias across countries [35].

### Statistical analysis

We used Mplus 8 to estimate all models with robust maximum likelihood estimation (MLR), which produces standard errors robust to non-normality of the data. Model fit was assessed according to the following criteria. First, we used the χ^2^ -statistic as offered by the MLR algorithm that is asymptotically equivalent to the Yuan–Bentler T2 test statistic [36]. The significance of this χ^2^ statistic indicated that the hypothesis of model exact fit to the data should be rejected. However, the rate of false mode rejection with large sample sizes and small degrees of model misspecification is high. Thus, we complemented the χ^2^ with other alternative goodness of fit indices, such as: the comparative fit index (CFI), the root mean square error of approximation (RMSEA), and the standardized root mean square residual (SRMR). The critical value of the chi-square is sensitive to large sample sizes and easily produces a statistically significant result [37]. Following Brown’s [38] recommendations, we accepted CFI values greater than .90, and RMSEA and SRMR (at both individual- and country-levels) values lower than .08.

As the difference between two scaled chi-squares for nested models is not distributed as a χ^2^, the tenability of the constraints imposed for testing measurement invariance was examined with the scaled difference chi-square [39]. Moreover, as the SBχ^2^ test has substantial power in large samples [37] we supplemented this statistic with the ΔCFI. In this regard, Cheung and Rensvold wrote that “it makes no sense to argue against the usefulness of the chi-square and rely on various goodness-of-fit indices (GFI) to evaluate the overall model fit, and then argue for the usefulness of the chi-square instead of various GFIs to test for measurement invariance” ([40], p. 252). Based on their simulation study, the authors recommended that investigators consider a difference in CFI larger than .01 as indicative of a meaningful change in model fit. Although we present both the SBχ^2^ and ΔCFI, we based our decisions about the equivalence of the models on the latter index, in accordance with the suggestion of Cheung and Rensvold [40]. Finally, following Jak [21], tests of significance of residual variances at the country level were conducted by using an alpha level of .01 instead of .05, to decrease the risk of capitalizing on chance. Moreover, the proportion of country-level bias in country-level variance (BIAS_coun_) and the proportion of country-level bias in total variance (BIAS_tot_) were estimated according to equations reported in Jak ([21], p. 81).

## Results

### Preliminary analyses

Before proceeding with the single-level CFA and MCFA, we examined the distribution of the BZSG scale items, in order to ascertain if there was any substantial deviation from normality. We found that skewness (*M* = .24, *SD* = .28) and kurtosis (*M* = -.72, *SD* = .23) were acceptable.

#### Step 1

As shown in Table 2, the hypothesized one factor model (i.e., M1) fitted to the entire sample resulted in an acceptable data fit. As can be seen, the hypothesized one-factor model showed a good fit. In Step 2, we examined ICCs for each of the eight BZSG scale items to determine whether the use of multilevel analysis was justified. As shown in the first column of Table 3, the ICCs ranged from .04 to .50, with an average of .14 (*SD* = .15), which is higher than the .10 criterion reported above.

**Table 2:** Single-level and multilevel CFA of BZSG scale.

Model	χ^2^	*df*	CFI	*p*	RMSEA	SRMR
M1. Single-level	1084.69	20	.959	< .01	.068	.031
M2. Within	449.26	20	.953	< .01	.044	-
M3. Between	94.44	20	.992	< .01	.018	-
M4. Multilevel configural	713.34	40	.926	< .01	.039	W = .031 B = .09
M5. Strong Isomorphism + zero between-level residuals	5268.10	55	.428	< .01	.093	W = .032 B = .38
M6. Strong Isomorphism + free between level residuals	769.91	47	.921	< .01	.037	W = .031 B = .18
	*Δ*χ^2^	*Δdf*	*Δ*CFI			
M5 vs M4	*	15	-.50			
M6 vs M4	18.06	7	-.01			

Note.

* This value could not be computed because the Scaling Correction Factor resulted undefined.

**Table 3:** ICC values from Step 2 and unstandardized and completely standardized factor loadings from Step 1 and Step 3 by BZSG scale items.

BZSG scale items		Single-level				Within				Between					
	*ICC*	*λ*_U_	*SE*	*p*	*λ*_CS_	*λ*_U_	*SE*	*p*	*λ*_CS_	*λ*_U_	*SE*	*p*	*λ*_CS_	*BIAS*_coun_	*BIAS*_tot_
1/ Successes of some people are usually failures of others.	.07	1.02	.02	< .01	.59	.97	.04	< .01	.59	.97	.04	< .01	.67	.56	.04
2/ If someone gets richer it means that someone else gets poorer.	.06	1.35	.01	< .01	.78	1.31	.05	< .01	.78	1.31	.05	< .01	.88	.22	.01
3/ Life is so devised that when somebody gains, others have to loose.	.10	1.37	.01	< .01	.79	1.30	.05	< .01	.79	1.30	.05	< .01	.76	.42	.04
4/ In most situations interests of different people are inconsistent.	.14	.58	.02	< .01	.39	.50	.03	< .01	.36	.50	.03	< .01	.30	.91	.11
5/ Life is like a tennis game—a person wins only when others lose.	.11	1.22	.02	< .01	.73	1.16	.04	< .01	.71	1.16	.04	< .01	.73	.47	.04
6/ When some people are getting poorer it means that other people are getting richer.	.50	1.30	.01	< .01	.78	1.25	.05	< .01	.77	1.25	.05	< .01	.97	.07	.00
7/ When someone does much for others he or she loses.	.11	0.75	.02	< .01	.46	.69	.04	< .01	.45	.69	.04	< .01	.43	.82	.07
8/ The wealth of a few is acquired at the expense of many.	.04	1.02	.02	< .01	.60	.99	.04	< .01	.59	.99	.04	< .01	.76	.42	.02
Factor Variance		*φ*_un_	SE	*P*	*φ*_cs_	*φ*_un_	SE	*p*	*φ*_cs_	*φ*_un_	SE	*p*	*φ*_cs_		
*φ*_1.2_						1	-	-	1.00	.092	.022	< .01	1.00		
Residual variance		θ_un_	SE	*p*	θ_cs_	θ_un_	SE	*p*	θ_cs_	θ_un_	SE	*p*	θ_cs_		
θ_1.1_		1.96	.03	< .01	.65	1.84	.08	< .01	.67	.11	.04	< .01	.56		
θ_2.2_		1.21	.03	< .01	.40	1.12	.09	< .01	.40	.05	.02	< .05	.22		
θ_3.3_		1.09	.03	< .01	.37	.99	.08	< .01	.37	.11	.05	< .05	.42		
θ_4.4_		1.89	.03	< .01	.85	1.69	.05	< .01	.87	.23	.05	< .01	.91		
θ_5.5_		1.41	.03	< .01	.47	1.34	.10	< .01	.50	.11	.02	< .01	.47		
θ_6.6_		1.10	.03	< .01	.39	1.07	.08	< .01	.41	.01	.01	< .05	.07		
θ_7.7_		2.07	.03	< .01	.79	1.90	.09	< .01	.80	.20	.04	< .01	.82		
θ_8.8_		1.88	.03	< .01	.64	1.80	.06	< .01	.65	.07	.023	< .01	.42		

*Notes*. ICC = intraclass coefficient; λU = Unstandardized loading estimate; λCS = completely standardized loading estimate. SE = Standard error; p = p-value; *φ*un = Unstandardized factor variance estimate; *φ*cs = completely standardized factor variance estimate; θun = Unstandardized residual estimate; θcs = completely standardized residual estimate

#### Step 2 and Step 3

In Steps 2 and 3 we compared, respectively, the fit of the model at the individual level (M2; within) and country level (M3; between; see Table 2). All in all, the proposed factor structure fitted the data very well at both levels of analysis. As can be seen in Table 3, all standardized factor loadings were significant, ranging at the individual level from .36 (item 4) to .79 (item 3), and at the country level from .72 (item 4) to .99 (item 2).

#### Steps 4–6

In these steps, tests of construct isomorphism for the BZSG scale across the individual and the country levels were conducted. As seen in Table 2, a two-level model with equal factor loadings and zero residual variance at the country level (M5; strong isomorphism) did not fit the data at all. Instead, the two-level model with equal factor loadings across levels and freely estimated residual variance at the between level shows good fit to the data (M6). This model was not significantly different from the baseline multilevel weak configural model (M4). Unstandardized and completely parameter estimates for this model, along with their respective standard errors are shown in Table 3.

According to our criterion for claiming statistical significance of the residual at the between-country level (i.e., *p* < .01), residuals associated with items 1, 4, 5, 7, and 8 (see Table 3) were significantly different from zero. According to Jak [20], these results suggest partial measurement invariance, and mean that the common factor is not the only factor influencing individual responses to these items at the country level. The proportions of country-level item variance bias ranged from .42 to .91 (*M* = .64; *SD* = .22), and the proportion of bias in the total item variances ranged from .02 to .11 (*M* = .06; *SD* = .04). Overall, the proportion of the overall variance in the latent BZSG at the between-country level was about 8%, whereas 92% of the BZSG variance was at the individual level.

#### Step 7

**Fig 1:**
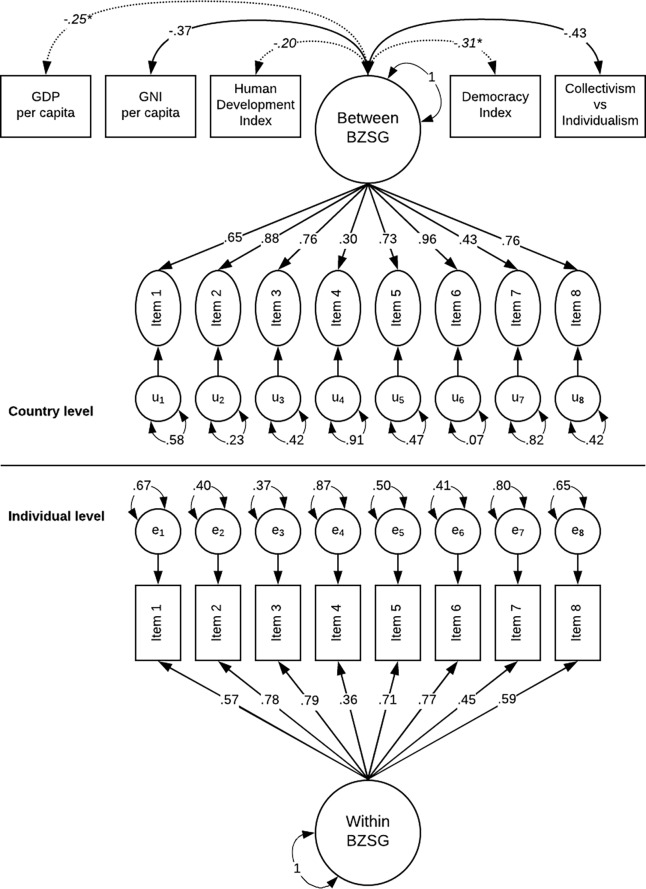
Two-level factor model BZSG items with the country-level variables: Gross domestic product per capita (GDP), Gross national income per capita (GNI), Human Development Index (HDI), Democracy Index (DEM. I.), and Collectivism-individualism (CO vs IN), with completely standardized parameter estimates.

At this point of the analyses, the country-level variables GNI per capita, GDP per capita, HDI, Democracy Index, and collectivism-individualism were included in the analyses as correlates of the country-level latent BZSG factor. This model showed an acceptable data fit χ^2^(81) = 1152.43, *p* < .01, CFI = .924, RMSEA = .034, SRMR_within_ = .031, SRMR_between_ = .166, and it is represented in Fig 1. All in all, only the GNI and the Individualism indices were significantly and negatively correlated with the latent factor of BZSG. The higher the scores in these indices, the lower the individuals’ scores on the latent BZSG. We also investigated the significance of any direct path from GNI per capita, GDP per capita, HDI, Democracy Index, and collectivism-individualism and the item residuals at the country level, but none of these paths resulted in significance.

## Discussion and conclusions

The aim of current study was to investigate the equivalence of the individual and the country level factor structure of the Belief in a Zero-Sum Game (BZSG) scale. Antagonistic beliefs about social relations was defined as describing zero-sum aspects of competition over scarce resources, which lead to intractable conflict between people or groups because it influences how individuals or nations perceive their relationship in situations where the interests are interdependent and there are limited resources. In this article, a test of construct isomorphism was conducted and relations between BZSG and other country and cultural dimensions, including macrosocial and macroeconomic indicators, were analyzed.

In cross-cultural research that focuses on multigroup comparisons, it is typically assumed that the measurement tool is operating in the same way, and that the underlying construct has the same factorial structure and conceptual meaning across the cultural groups. In our study, we found evidence for a strong isomorphism for the BZSG scale with non-zero factor residual scale. Thus, we conclude that individual and country differences in BZSG scores have the same psychological meaning–i.e., it is an isomorphic construct across levels: as an individual zero-sum mindset and as a cultural worldview ideology. At the individual level, individuals fight over limited resources, and a similar phenomenon exists at the societal level, as reflected in a generalized national hostility towards out-groups. These findings are consistent with realistic-group-conflict theory, which proposes that perceived group competition for resources leads to inter-group competition for scarce resources such as land or jobs [41] or negative attitudes towards immigration [42]—although immigrants are often essential to the functioning of first-world economies [43]. Also, the finding that social antagonism appears at a societal level is consistent with Foster’s studies [44] about peasant societies and compatible with results showing that BZSG is more prevalent among members of rural societies with lower socio-economic standing. Our conclusions about isomorphism are consistent with the theory of social axioms [9], which describes general social beliefs as interpreting culture and the behavior of individuals socialized into those cultures.

In our investigation of the relationship between BZSG at the country level and other macro indicators, collectivism-individualism and national income [13] were once again significant correlates in two-level one-factor model BZSG items with the country-level variables across levels. What is important is that this time we measure income through GNI per capita index, as it is better measure of the standard of living than GDP, which only measures production. What more, GNI per capita (taking into account the GDP per capita and HDI), is still significantly correlated with BZSG at the country level.

Our results confirm that BZSG emerges in hierarchical collectivist societies with lower income. The key seems to be the level of interconnectedness: in societies with tight relations and network obligations, resources are perceived as limited, so citizens feel validated for fighting for resources.

The link between socio-economic systems and psychological processes allows for the possibility of finding ways to shape international relations by influencing individuals. As previous studies confirmed that zero-sum thinking appears to change as individuals learn more about the nonzero-sumness of a situation [45,46], it provides the opportunity for intervention in education at the individual level. Burleigh [47] claims that to the extent that zero-sum thinking promotes antagonistic “cut-throat” behavior or non-cooperation that is contrary to pedagogical outcomes, it is important to address the sources of zero-sum thinking in the classroom and promote an awareness of nonzero-sum possibilities for interaction. Such intervention at the individual level could bring cultural outcomes, including more balanced international relations or changes in global mentality towards joint profit exchange. Wright [48] has already postulated that the creation of new forms of nonzero-sum individual interaction (“potential synergy”) drive cultural progress and societal welfare. At the societal level, social and economic system transformation could be implemented in national developmental programs and community-level action.

BZSG seems to be a useful concept in characterizing and understanding individuals and cultures, a promising candidate for explaining variance in cross-cultural differences that are not readily accounted for by values—where culture may influence the way axioms operate to influence psychological outcomes of individuals [8] or individuals can influence national attitudes. Work clarifying the conditions and consequences of BZSG is very important, as knowledge about social axioms (as cultural descriptors which guide behavior of individuals in a culture), is crucial for the sociocultural adaptation of immigrants [49].

An important limitation of the study reported here is that the sample was composed solely of students and cannot be considered representative of any society as a whole. However, the data do provide an indication of the direction of young elites. Another limitation is the sample size at the country level, which was high in terms of cultures covered, but not especially high from a pure statistical standpoint, and this may have had an impact on the reliability of standard errors [50].

Future studies should consider the use of representative samples from around the world to better investigate the construct isomorphism of the BZSG scale. Also, after establishing equivalence at the individual and country levels, individual-level models of social antagonism across national cultures could be developed. However, our results present the possibility of applying the BZSG scale in cross-cultural studies in at least 43 countries.
